# Improved control effect of pathological oscillations by using delayed feedback stimulation in neural mass model with pedunculopontine nucleus

**DOI:** 10.1002/brb3.3183

**Published:** 2023-08-02

**Authors:** Yingpeng Liu, Rui Zhu, Ye Zhou, Jiali Lü, Yuan Chai

**Affiliations:** ^1^ School of Mathematics and Physics Shanghai University of Electric Power Shanghai China

**Keywords:** delayed feedback stimulation, neural mass model, Parkinson's disease, pedunculopontine nucleus

## Abstract

**Background:**

The role of delayed feedback stimulation in the discussion of Parkinson's disease (PD) has recently received increasing attention. Stimulation of pedunculopontine nucleus (PPN) is an emerging treatment for PD. However, the effect of PPN in regulating PD is ignored, and the delayed feedback stimulation algorithm is facing some problems in parameter selection.

**Methods:**

On the basis of a neural mass model, we established a new network for PPN. Four types of delayed feedback stimulation schemes were designed, such as stimulating subthalamic nucleus (STN) with the local field potentials (LFPs) of STN nucleus, globus pallidus (GPe) with the LFPs of Gpe nucleus, PPN with the LFPs of Gpe nucleus, and STN with the LFPs of PPN nucleus.

**Results:**

In this study, we found that all four kinds of delayed feedback schemes are effective, suggesting that the algorithm is simple and more effective in experiments. More specifically, the other three control schemes improved the control performance and reduced the stimulation energy expenditure compared with traditional stimulating STN itself only.

**Conclusion:**

PPN stimulation can affect the new network and help to suppress pathological oscillations for each neuron. We hope that our results can gain an insight into the future clinical treatment.

## Graphical abstract

A new network with pedunculopontine nucleus based on a neural mass model was established. Four types of delayed feedback stimulation schemes were designed. Suppression of pathological oscillations for each neuron by the stimulation of pedunculopontine nucleus was revealed.

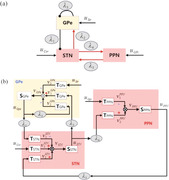

## 1 INTRODUCTION

Parkinson's disease (PD) including sleep, cognitive, and motor disorders is a neurodegenerative disease (Delaville et al., 2014; Elfil et al., 2021). Clinical features in PD show the broken function of dopaminergic neurons in the substantia nigra, which can synthesize and release the neurotransmitter dopamine (DA) (Corti et al., 2011; Heo et al., 2017), thus leading to a significant reduction of DA content in the striatum (Brammerloh et al., 2021).

Many researchers suggested that stimulation targeting subthalamic nucleus (STN) and globus pallidus interna (GPi) (Hou et al., 2022) can improve the motor symptoms of patients. However, they usually ignore the role of globus pallidus (GPe) in the central brain region of the basal ganglia, and stimulating GPe may have a similar effect. The mechanism of improving disease remains unclear, but one possibility is stimulating the STN and GPi to improve PD through GPe (Qiu et al., 2016). The STN has excitatory projections to GPe, GPe has inhibitory projections to STN, and GPe has inhibitory projections to GPi (Chen et al., 2015). Clinical studies indicated that GPe injury in monkeys can worsen PD (Zhang et al., 2006). Abnormal GPe neuronal activity is strongly associated with motor abnormalities in patients with PD. Given that GPe in the central brain region of the basal ganglia may be another important stimulation target for PD treatment (Kopell et al., 2006; Lourens et al., 2011). Recently, experiments have identified that the activation of GPe can directly affect the activity of thalamic neurons through its inhibitory projections in thalamic reticular nucleus and can change the activity of the projections to the striatum and influence the pattern of GPi by the projections from STN to GPe (Hahn et al., 2008; Vitek et al., 2004). Therefore, these GPe‐related pathways may contribute to improvement in patients with PD when GPe is applied with electrical stimulation (Johnson et al., 2012; Kumaravelu et al., 2016). For the side effects caused by the stimulation of the GPe region, we can reduce the stimulation parameters to alleviate the side effects and use drugs to cooperate with the treatment (Budman et al., 2018; Castillo et al., 2020). These studies show that the activity pattern and rate of all neuron nodes in the basal ganglia thalamic network were changed (Vitek et al., 2012), and stimulating GPe may be as effective as GPi in controlling PD with motor symptoms (Qiu et al., 2016).

More and more researchers use the neural mass model to study and analyze neural dynamics. Basu et al. (2018) predicted the human brain's response to different frequencies of electrical stimulation and explored larger parameter space using less experimental data by the neural mass model. Recently, Liu et al. (2019) established a new neural mass model, which shows stimulating STN and GPe can suppress the pathological oscillation activities in the STN‐GPe network. The parameters of the neural mass model are lesser than those of the more detailed biophysical models. The modeling method of the neural mass model is a compromise between detailed modeling and abstract modeling and provided biophysically interpretable results (Song et al., 2019). The neural mass model has been widely used in the study of PD, which can explain the death of DA neurons and the decrease of the DA level in the brain of patients, resulting in the remodeling and functional changes of neuronal networks, thus the appearance of PD symptoms (Basu et al., 2018; Jimenez et al., 2013). Therefore, by changing these coupling strengths between STN, GPe, and pedunculopontine nucleus (PPN) neurons and the self‐coupling strength of GPe, we proposed a new neural mass model to reappear the parkinsonian symptoms (beta oscillations).

However, the effect of PPN has not taken into account based on the neural mass model in previous studies. PPN is considered to be a potential stimulation target for patients with PD (Dayal et al., 2021; Galazky et al., 2018). Thevathasan et al. (2018) introduced that DBS in PPN is a novel and promising therapeutic approach, and the PPN target is mainly suitable for drug‐resistant gait freezing. Ferraye et al. (2011) suggested that PPN stimulation specificity is better than STN in the treatment of gait disorders. Recently, Molina et al. (2021) proposed a new closed‐loop stimulation method for PPN, and 60% of subjects showed some improvement in freezing of gait (FoG) within 6 months. Meanwhile, deep brain stimulation (DBS) of PPN can improve axial motor disorders in PD (Hamani et al., 2016; Mori et al., 2016; Y. Yu et al., 2019), especially gait freezing and falling symptoms (Huang et al., 2018; K. Yu et al., 2020). Considering previous studies on PPN, we established a new model based on the neural mass model for PPN. In our study, the stimulation of PPN can suppress beta frequency oscillatory activity and is a complement to the previous model.

Impaired PPN function in patients may result in motor and non‐motor symptoms. By training mice to run, Li and Spitzer (2020) found that PPN neurons were heavily activated. Cholinergic neurotransmitter conversion to gamma‐aminobutyric acid (GABA) occurs in the PPN region of the brain during exercise, which promotes the exercise ability. The PPN acts as a relay station to receive outgoing motor information from the cerebral cortex and transmit this signal to the motor areas of the thalamus, brain stem, and spinal cord. In addition, the connection of PPN to basal ganglia may also activate PPN neurons to improve motor symptoms (Rauch et al., 2010). In addition, PPN‐DBS can improve the non‐motor symptoms of nocturnal sleep. The muscarinic receptors of PPN are activated, rebalancing sleep physiology because electrical stimulation alters the functional activity of the cholinergic neuronal population (Anderson et al., 2017). By incorporating the neural mass model, we can understand that the PPN firing pattern, neurotransmitter release, and connectivity contribute to the emergence and progression of motor and non‐motor symptoms. In addition, we can better explore the interaction between PPN and other brain regions. This approach may lead to the identification of new therapeutic avenues and provide a more comprehensive understanding of the disease.

**FIGURE 1:**
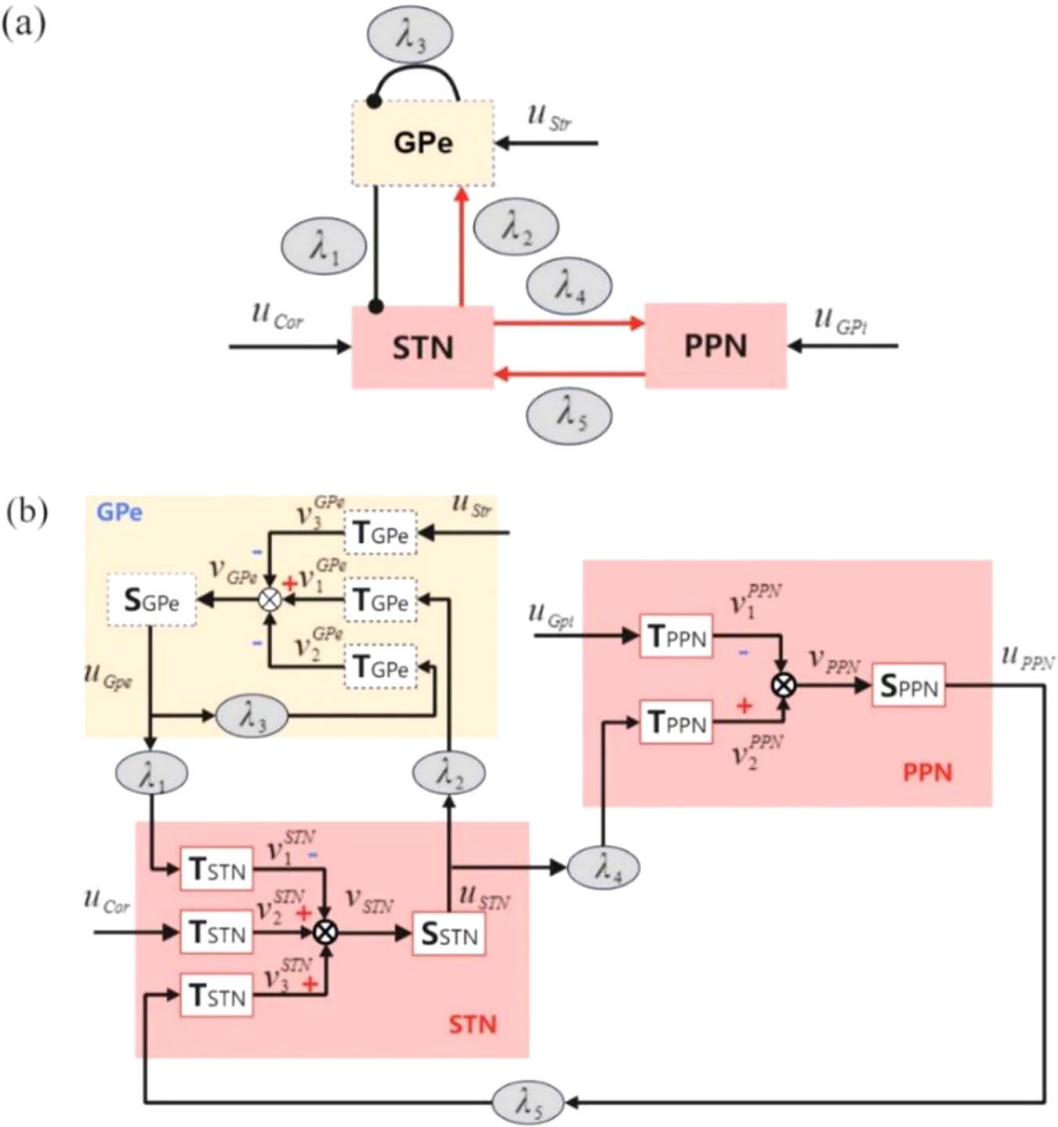
Framework of the new neural mass model. (a) Major functional connections of the neural mass model. (b) Detailed connections between of the three neural populations. The network contains three nuclei: subthalamic nucleus (STN), globus pallidus (GPe), and pedunculopontine nucleus (PPN). The red lines with arrowheads represent the excitatory projection of glutamate receptors, and the black lines with round heads indicate the GABA‐mediated inhibitory projections, where these nuclei corresponding coupling strengths areλ_1_,λ_2_,λ_3_,λ_4_, andλ_5_. In this model,$S_{i}\left(i\in \left\{\mathrm{STN},\mathrm{GPe},\mathrm{PPN}\right\}\right)$are nonlinear blocks and$T_{i}\left(i\in \left\{\mathrm{STN},\mathrm{GPe},\mathrm{PPN}\right\}\right)$are linear blocks. The average pulse density of action potentials$u_{i}\left(i\in \left\{\mathrm{STN},\mathrm{GPe},\mathrm{PPN}\right\}\right)$and an average postsynaptic membrane potential$v_{i}\left(i\in \left\{\mathrm{STN},\mathrm{GPe},\mathrm{PPN}\right\}\right)$can transform into each other.*u*_Str_,*u*_Cor_, and*u*_GPi_denote the noisy input from the striatum (Str), cortex (Cor), and GPi, respectively.

Pahapill (2000) analyzed the link between PPN and PD from an anatomical and physiological perspective. The PPN is mainly divided into two large groups of neurons: the pars dissipatus of PPN (PPNd) is the main region in PPN and scattered throughout the PPN region, and the pars compacta of PPN (PPNc) is mainly clustered in the caudal side of PPN (French & Muthusamy, 2018). The main neurotransmitters that can affect the firing of PPN neurons are glutamate, acetylcholine, and GABA, and studies have shown that the cholinergic PPN activity can be activated by injecting a concentration of glutamate into the PPN area, thereby improving locomotion. However, acetylcholine GABA inhibits the PPN activity and leads to dyskinesia. The PPNc mainly contains cholinergic and glutaminergic neurons, and PPNd mainly contains GABAergic neurons. The glutamate is projected from STN to PPN, PPN has cholinergic nucleus and non‐cholinergic projection to STN, and there is reciprocal excitatory projection between PPN and STN. The STN has excitatory projection to GPe, GPe has inhibitory projection to STN, and GPe has self‐inhibitory projection (Y. Yu et al., 2019). Figure 1 shows the connections between PPN, STN, and GPe neurons.

Delayed feedback stimulation is an effective way to suppress and solve beta oscillations, which belongs to close‐loop control schemes (Dovzhenok et al., 2013; Hauptmann et al., 2005). Pyragas (1992) successfully applied the delayed feedback control in chaotic system to interrupt synchronization for the first time. H. Yu et al. (2013) pointed out that traditional deep brain stimulations (DBSs) have many limitations, such as determining stimulation parameters. In addition, they studied linear delayed feedback stimulation using the collective population signal to suppress collective synchronization. However, previous researchers have made tremendous efforts in optimizing delayed feedback algorithm such as parameter selection and energy expenditure (Popovych & Tass, 2019; H. Yu et al., 2013). In addition, they were used to improve the control effect of the algorithm or only stimulate STN itself in most algorithms, rather than using stimulation with feedback from other nuclei (Popovych & Tass, 2018; Rosenblum & Pikovsky, 2004). To address the question, and considering the importance of GPe and PPN, we proposed three delayed feedback stimulation schemes involving GPe and PPN compared with previous stimulating STN itself.

Traditional closed‐loop stimulation mainly focuses on the nucleus of the stimulated target, most of which stimulates STN itself. Recent clinical experiments have demonstrated that DBS in GPe with a high frequency has positive regulation in PD (Johnson et al., 2012; Kumaravelu et al., 2016). In PD, STN DBS has been found to regulate the activity of other relevant regions, such as the pallidum and motor cortex (Mandali et al., 2015; Prabhu et al., 2015). This suggests that the therapeutic effects of DBS can extend beyond the site of direct stimulation. While the initial focus may be on specific target nuclei, the adaptability of CL‐DBS allows for broader effects beyond the site of immediate stimulation. Hauptmann et al. (2005) stimulated the target population with the local field potentials (LFP) of other population sites to achieve desynchronization of the network. Few studies have used delayed feedback stimulation in the neural mass model. Previous research has shown that basal ganglia is considered a major brain structure to suppress the PD and that the connection between the basal ganglia and other brain neurons is ignored. However, PPN neurons injury may lead to motor dysfunction in PD, which mostly can be improved by low frequency stimulation, suggesting that PPN is a target for improving the disease (French & Muthusamy, 2018; Thevathasan et al., 2018). In order to understand the role of PPN in suppressing PD, Y. Yu et al. (2019) proposed a new BGCT‐PPN model over the previous BGCT network and mainly studied the projection from PPN to STN to suppress PD, which demonstrated this pathway can inhibit pathological oscillations in PD through this computational model. Therefore, we designed four delayed feedback stimulus schemes to explore the relationship between PPN, STN, and GPe in the neural mass model.

Using a series of analysis techniques, we showed that stimulating STN, GPe, and PPN can suppress the beta oscillatory in neural mass model and PPN system. Meanwhile, in the process of simulation experiment, we researched that all of the control strategies designed are effective, and the other three novel strategies work better than stimulating STN alone. Therefore, our simulation results provide theoretical support for future clinical trials, which may provide the diversity of stimulation modes and selectivity of stimulation targets. Consider that only a small percentage of patients actually use PPN DBS in clinical treatment. In this study, “suppresses PD” means suppress the PD with FoG and drug resistance.

## 2 MATERIALS AND METHODS

### 2.1 Neural mass model

Considering the STN‐GPe circuits in the BG and PPN for suppressing the pathological oscillatory activity (Alekhya & Chakravarthy, 2015; Y. Yu et al., 2019), we established a new neural mass model (Liu et al., 2017) to reveal the feature in pathological oscillatory state, inspired by the model used successfully in basal ganglia‐cortico‐thalamic and cortex (Cor). As shown in Figure 1, the new model consists of three neural populations, which are STN, GPe, and PPN. STN receives excitatory input from PPN and synaptic inhibition from GPe. GPe receives inhibition from itself and excitatory projection from STN. PPN has excitatory projection from STN. Besides, the excitatory projections$u_{Cor}$and inhibitory projections$u_{Gpi}$and $u_{Str}$represent the independent noisy external signals. In this model, the action potentials$u_{i}$and membrane potential$v_{i}$can convert to each other by a nonlinear block$S_{i}$and a linear block$T_{i}$. Meanwhile, the value of$v_{i}$can be viewed as the LFPs (Basu et al., 2018; Bhattacharya et al., 2016).

### 2.2 Basic equations and evaluation index

To describe better the dynamical phenomenon in the neural mass model, the sigmoid functions model the nonlinear elements, and the second‐order transfer functions model the linear elements. Furthermore, nonlinear block$T_{i}\left(i\in \left\{STN,GPe,PPN\right\}\right)$corresponding formula is$T_{i}=H_{i}x_{i}/{\left(x_{i}s+1\right)}^{2}$, where$x_{i}$and$H_{i}$are the parameters in linear block and*s*is the Laplace variable (s=σ+jω). In previous studies, transfer function describing the relationship between the inputs and outputs of linear systems can be transferred into a differential equation corresponding to the system. We obtained $x_{i}^{2}s^{2}V\left(s\right)+2x_{i}sV\left(s\right)+V\left(s\right)=H_{i}x_{i}U\left(s\right)$from$T_{i}=V\left(s\right)/U\left(s\right)=H_{i}x_{i}/{\left(x_{i}s+1\right)}^{2}$ by rewriting the transfer function. By applying the operatordv/dtinstead of Laplace operator*s*, thedv/dtcan be rewritten as$v^{\prime \prime }.$Therefore, we further obtained $v^{\prime \prime }=H_{i}/x_{i}u-2/x_{i}v^{\prime }-1/x_{i}^{2}v\left(i\in \mathrm{STN},\mathrm{GPe},\mathrm{PPN}\right)$. The math equation of the system could be described as follows:

For the STN

(1) $$v_{1}^{\prime \prime \mathrm{STN}}=\frac{\lambda_{1}H_{s}}{x_{s}}u_{\mathrm{GPe}}-\frac{2}{x_{s}}v_{1}^{\prime \mathrm{STN}}-\frac{1}{x_{s}^{2}}v_{1}^{\mathrm{STN}},$$

(2) $$v_{2}^{\prime \prime \mathrm{STN}}=\frac{H_{s}}{x_{s}}u_{\mathrm{Cor}}-\frac{2}{x_{s}}v_{2}^{\prime \mathrm{STN}}-\frac{1}{x_{s}^{2}}v_{2}^{\mathrm{STN}},$$

(3) $$v_{3}^{\prime \prime \mathrm{STN}}=\frac{\lambda_{5}H_{s}}{x_{s}}u_{\mathrm{PPN}}-\frac{2}{x_{s}}v_{3}^{\prime \mathrm{STN}}-\frac{1}{x_{s}^{2}}v_{3}^{\mathrm{STN}},$$

(4) $$v_{\mathrm{STN}}=v_{3}^{\mathrm{STN}}+v_{2}^{\mathrm{STN}}-v_{1}^{\mathrm{STN}}.$$

For GPe

(5) $$v_{1}^{\prime \prime \mathrm{GPe}}=\frac{\lambda_{2}H_{g}}{x_{g}}u_{\mathrm{STN}}-\frac{2}{x_{g}}v_{1}^{\prime \mathrm{GPe}}-\frac{1}{x_{g}^{2}}v_{1}^{\mathrm{GPe}},$$

(6) $$v_{2}^{\prime \prime \mathrm{GPe}}=\frac{\lambda_{3}H_{g}}{x_{g}}u_{\mathrm{GPe}}-\frac{2}{x_{g}}v_{2}^{\prime \mathrm{GPe}}-\frac{1}{x_{g}^{2}}v_{2}^{\mathrm{GPe}},$$

(7) $$v_{3}^{\prime \prime \mathrm{GPe}}=\frac{H_{g}}{x_{g}}u_{\mathrm{Str}}-\frac{2}{x_{g}}v_{3}^{\prime \mathrm{GPe}}-\frac{1}{x_{g}^{2}}v_{3}^{\mathrm{GPe}},$$

(8) $$v_{\mathrm{GPe}}=v_{1}^{\mathrm{GPe}}-v_{2}^{\mathrm{GPe}}-v_{3}^{\mathrm{GPe}}.$$

For the PPN

(9) $$v_{1}^{\prime \prime \mathrm{PPN}}=\frac{H_{p}}{x_{p}}u_{\mathrm{Gpi}}-\frac{2}{x_{p}}v_{1}^{\prime \mathrm{PPN}}-\frac{1}{x_{p}^{2}}v_{1}^{\mathrm{PPN}},$$

(10) $$v_{2}^{\prime \prime \mathrm{PPN}}=\frac{\lambda_{4}H_{p}}{x_{p}}u_{\mathrm{STN}}-\frac{2}{x_{p}}v_{2}^{\prime \mathrm{PPN}}-\frac{1}{x_{p}^{2}}v_{2}^{\mathrm{PPN}},$$

(11) $$v_{\mathrm{PPN}}=v_{2}^{\mathrm{PPN}}-v_{1}^{\mathrm{PPN}}.$$

Based on a series of equations mentioned above, similar to previous work (Liu et al., 2019), the relationship between $u_{i}\left(i\in \left\{\mathrm{STN},\mathrm{GPe},\mathrm{PPN}\right\}\right)$and $v_{i}\left(i\in \left\{\mathrm{STN},\mathrm{GPe},\mathrm{PPN}\right\}\right)$can be described as the sigmoid function:

(12) $$u_{\mathrm{STN}}=S_{\mathrm{STN}}\left(v_{\mathrm{STN}}\right)=\frac{r_{s}}{1+e^{-y_{s}\left(v_{\mathrm{STN}}+w_{s}\right)}},$$

(13) $$u_{\mathrm{GPe}}=S_{\mathrm{GPe}}\left(v_{\mathrm{GPe}}\right)=\frac{r_{g}}{1+e^{-y_{g}\left(v_{\mathrm{GPe}}+w_{g}\right)}},$$

(14) $$u_{\mathrm{PPN}}=S_{\mathrm{PPN}}\left(v_{\mathrm{PPN}}\right)=\frac{r_{p}}{1+e^{-y_{p}\left(v_{\mathrm{PPN}}+w_{p}\right)}},$$

where$y_{j}\left(j\in \left\{\mathrm{STN},\mathrm{GPe},\mathrm{PPN}\right\}\right)$ and $r_{j}\left(j\in \left\{\mathrm{STN},\mathrm{GPe},\mathrm{PPN}\right\}\right)$ are the parameters of the sigmoid function. The $u_{j}\left(j\in \left\{\mathrm{STN},\mathrm{GPe},\mathrm{PPN}\right\}\right)$ represented four kinds of delayed feedback stimulation signals. We listed the values of the parameters used in Table 1, where these parameters were referred to (Liu et al., 2019), and the letters *s*, *g*, and *p* stood for STN, GPe, and PPN, respectively.

**TABLE 1:** Default parameter values.

Parameters	Values
$H_{s}$	20
$H_{g}$	20
$H_{p}$	20
$x_{s}$	0.006
$x_{g}$	0.014
$x_{p}$	0.005
$r_{s}$	300
$r_{g}$	400
$r_{p}$	200
$y_{s}$	0.1
$y_{p}$	0.1
$y_{g}$	0.1
λ_1_	1.12(Normal)−5(Pathological)
λ_2_	19*(Normal)−20(Pathological)
λ_3_	6.6(Normal)−2(Pathological)
λ_4_	10(Normal)−13(Pathological)
λ_5_	3(Normal)−1(Pathological)
*x*_Cor_	27+0.1*randn(n)
*x*_Str_	2+0.1*randn(n)
*x*_GPi_	20+0.1*randn(n)

All stimulations were accomplished in the MATLAB R2018a (Math Works) environment. The equations were solved by the forward Euler scheme, and the stimulation time was 3 s with time step 0.0001 s. To ensure the accuracy of the results, we carried out all the simulations 20 times.

In this study, based on Liu et al. (2019), three evaluation indexes were established to evaluate the effectiveness of four stimulation schemes and the oscillatory network states.

(1) *Power spectra*: The power spectra were estimated by calculating fast Fourier transform for the LFPs of each nucleus. The dominant frequency was described by the maximum peak frequency, and the corresponding power of the oscillation activities was calculated. To remove the direct current component, power spectra were obtained by subtracting the average LFPs.

(2) *Oscillation index* $\left(O_{I}\right)$: In order to quantitatively observe the level of oscillation with adding stimulation, oscillation index was used to evaluate the effect of suppressing pathological oscillation. In addition, the small value of oscillation index represents was effective for suppressing PD. In addition, to exclude the disturbance of the transient and quantify the oscillatory state, we calculated the standard deviations of the LFPs in the last second for all nuclei including STN, GPe, and PPN after adding the stimulation. Besides, N=3/dt is the LFPs data of 3 s.

(15) $$O_{I}^{i}=\sqrt{\frac{\sum {}_{2/dt+1}^{N}{\left(v_{i}-\mathrm{mean}\left(v_{i}\right)\right)}^{2}}{1/dt}}.i\in \left(\left\{\mathrm{STN},\mathrm{GPe},\mathrm{PPN}\right\}\right)$$

(3) *Energy index* $\left(E_{I}\right)$: To comprehensively evaluate the energy consumption of four stimulation schemes, energy index was defined as the average stimulation power. We added stimulation at the beginning of the 2nd s to estimate the control effect, thus bringing the time of energy consumption over 2 s.

(16) $$E_{I}=\frac{\sum {}_{1/dt+1}^{N}w_{i}^{2}}{2/dt}.$$

### 2.3 Delayed feedback control strategies

**FIGURE 2:**
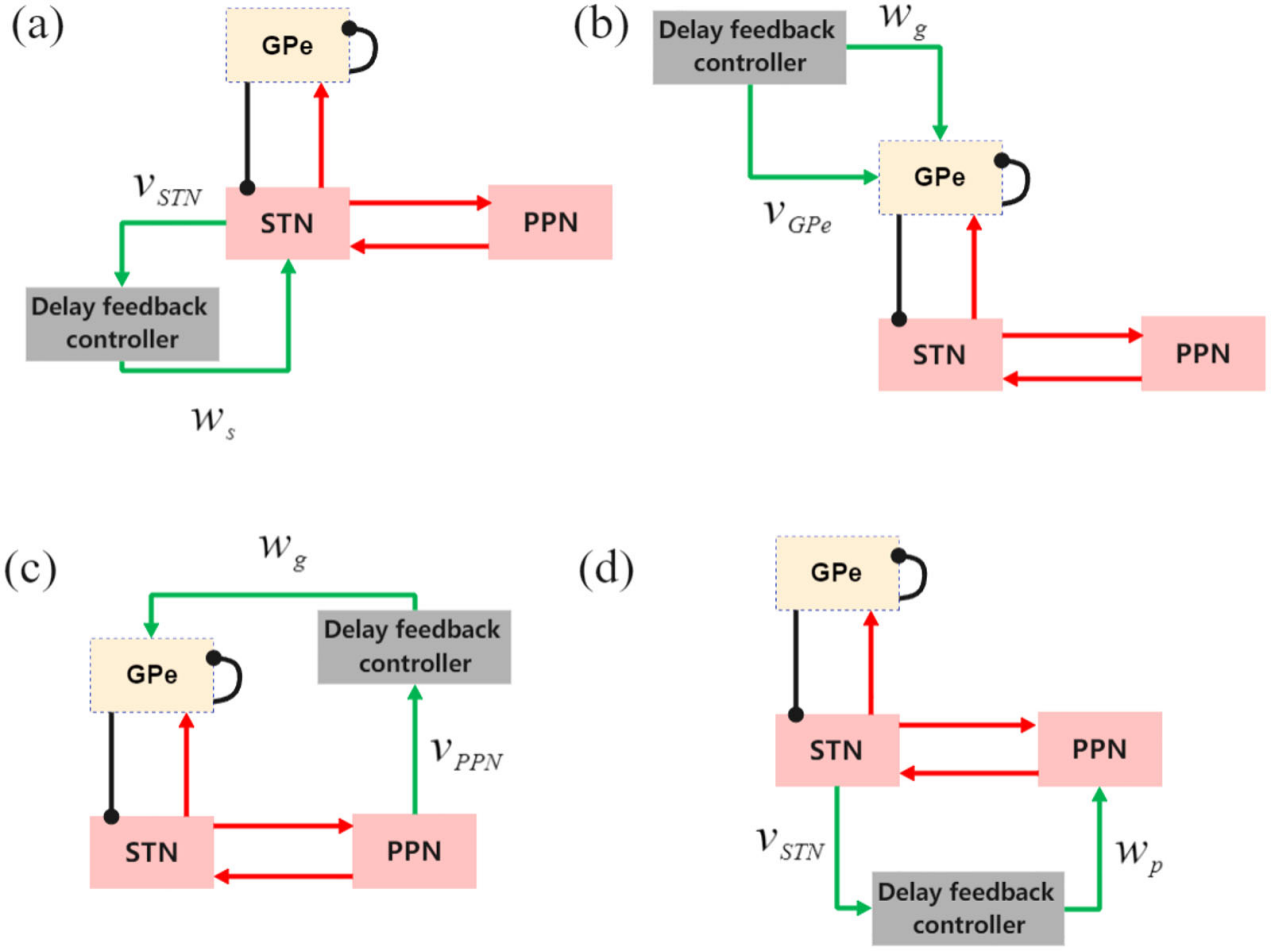
Strategy patterns of the four delayed feedback control: (a‒d) Control strategies (a‒d).

Previous studies have successfully confirmed that delayed feedback is an effectively desynchronizing control scheme and ameliorates symptoms with an abnormal neuronal synchronization characteristic. In this study, we applied four delay feedback stimulations patterns considering an interaction between STN, GPe, and PPN (as shown in Figure 2) to suppress PD into a normal state and interrupt synchronization. In Figure 2, we stimulate the nucleus with the control signal of the LFPs of the other nuclei, including the traditional project to stimulate the STN itself and three novel projects. The stimulation signal (Popovych & Tass, 2018; Pyragas et al., 2007) $w_{m}\left(m\in \left\{STN,GPe,PPN\right\}\right)$can be calculated from LFPs of the corresponding nucleus, and the algorithm can be described as

(17) $$w_{m}\left(t\right)=K\left(v_{n}\left(t-\tau \right)-v_{n}\left(t\right)\right),$$

where*K*denotes the stimulation intensity andτdenotes the time delay.

## 3 RESULTS

### 3.1 Normal and pathological states

**FIGURE 3:**
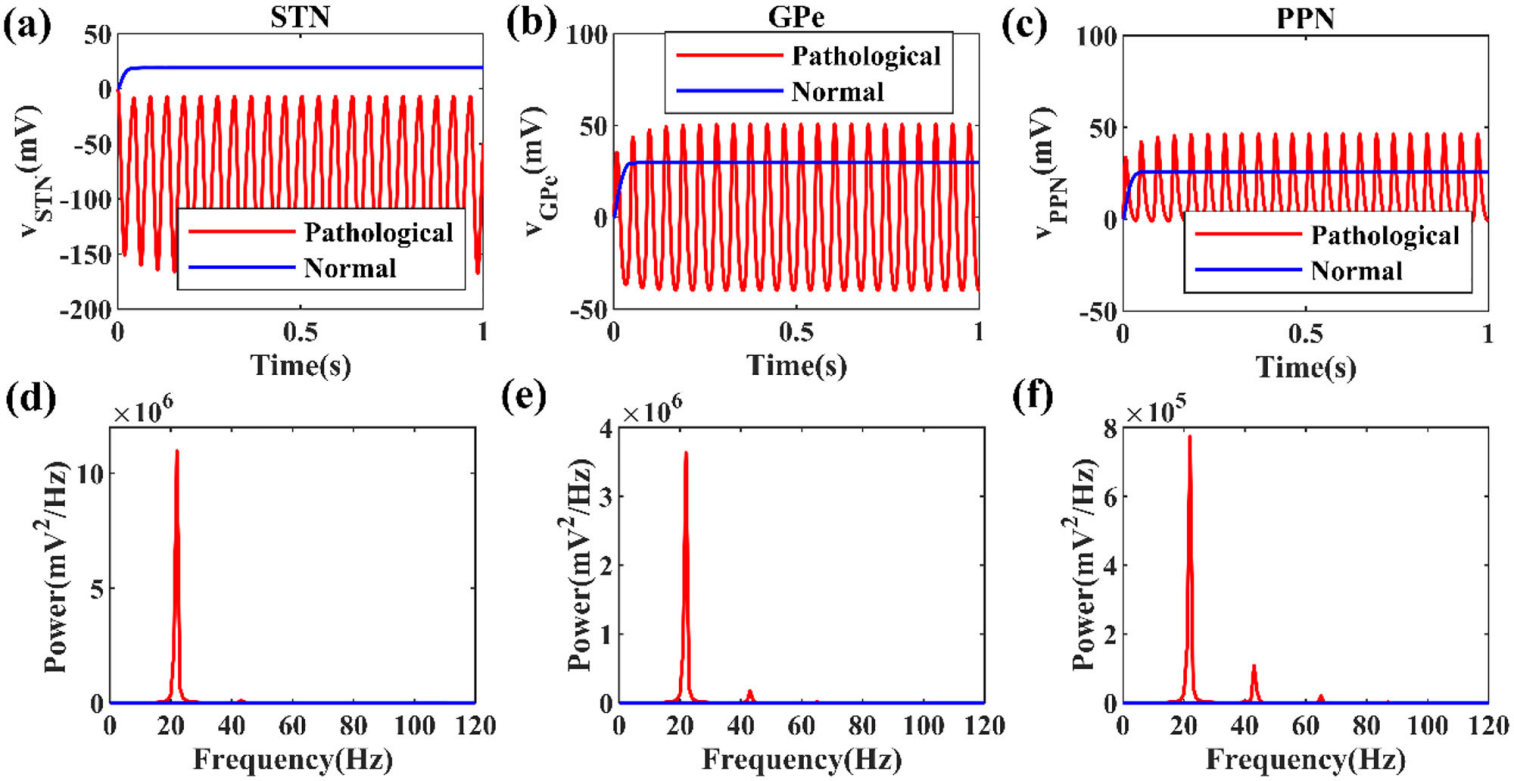
The time series of local field potentials (LFPs) in normal and pathological states corresponding to the neural population (a) subthalamic nucleus (STN), (b) globus pallidus (GPe), and (c) pedunculopontine nucleus (PPN). The frequency and their corresponding power in normal and pathological states are shown in (d), (e), and (f).

The activities of STN, GPe, and PPN in the neural mass model can appear the pathological and normal states by modifying coupling strengths among the three nuclei (i.e., λ_1_,λ_2_,λ_3_,λ_4_, andλ_5_shown Figure 1 and Table 1), which present the possible changes in physiology with the DA changes. For the coupling strengths choices, the parameter is within the physiological range and modest. Figure 3a‒c illustrates the enhanced oscillatory activities of all STN, GPe, and PPN compared with normal conditions. As shown in Figure 3d‒f, the power spectra are used to analyze the dominant oscillation frequency around 22 Hz. The power spectra of three nuclei increase significantly in beta frequency peak for the pathological states.

### 3.2 Effect of four delayed control schemes

The model consists of three nuclei, which can reproduce pathological and normal states before added stimulation. In the pathological state, STN, GPe, and PPN fire bursts of action potentials at a frequency of 22 Hz because of their strong excitatory and inhibitory connections (Magill et al., 2000).

**FIGURE 4:**
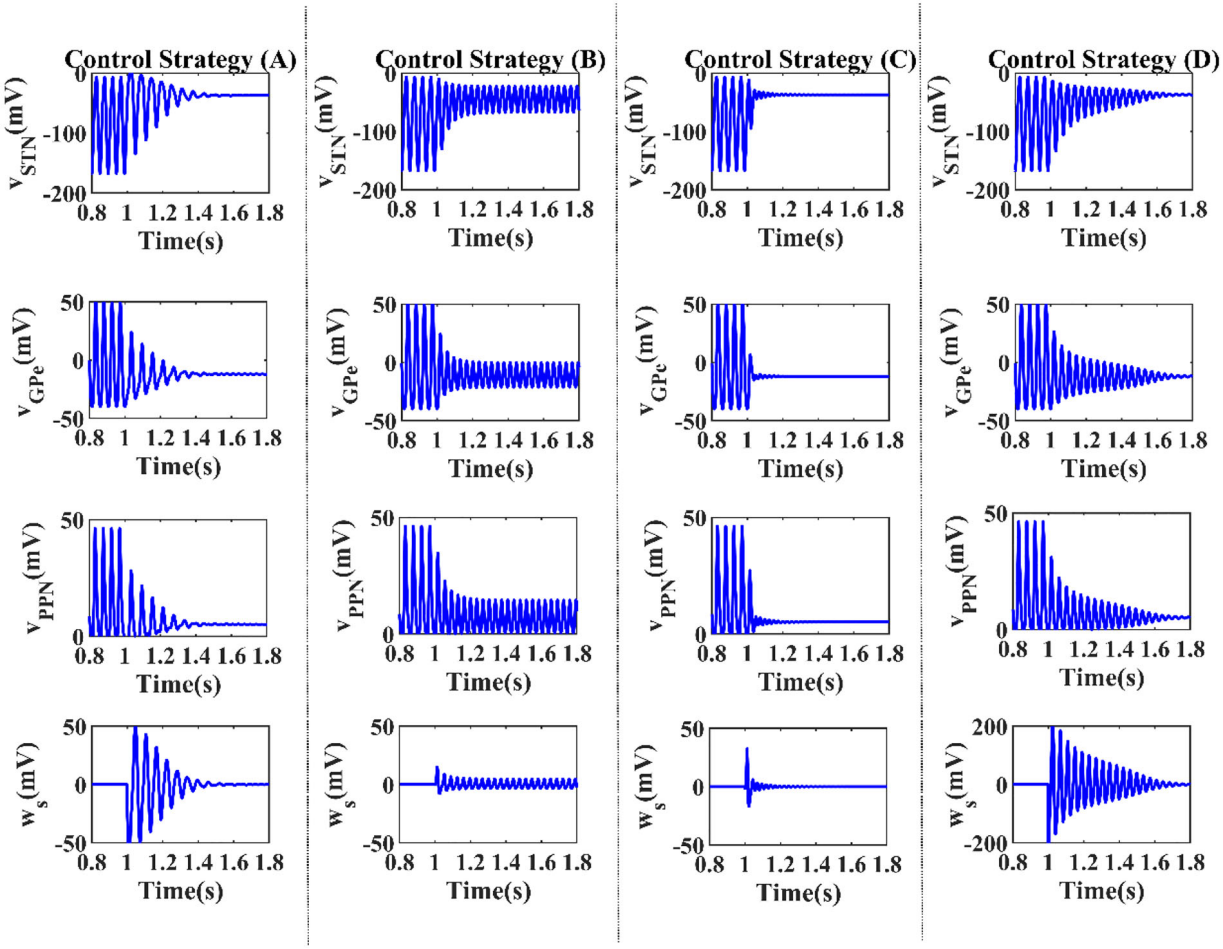
Control effect of four types of delayed feedback control schemes. All feedback stimulations are added att=1s. Control strategies (a–d) are(K,τ)=(0.4,25ms), (K,τ)=(−2,2ms),(K,τ)=(−2,4ms), (K,τ)=(3,8ms).

Figure 4 shows the control effect of four types of delayed feedback stimulation schemes. The delayed feedback stimulation scheme often uses the time delay with half of the oscillatory excitatory (Popovych et al., 2017). However, the ordinary time delay seems not to be an optimal choice in our computational model. Therefore, time delay was first adjusted toτ=25msin control strategy (A). Meanwhile, control strategies (B–D) also adjusted to the parameterτ=2ms,τ=4ms, andτ=8ms, respectively. To suppress the pathological state, the stimulation intensity in schemes (A) and (D) was set to *K* = 0.4 and *K* =3, respectively, and schemes (B) and (C) were both set to *K* = ‒2.

For these parameter selections, suppressing the pathological activities while preserving its beta frequency in the pathological state around 22 Hz, the parameter *K* andτare modest and appropriate. Four delayed feedback stimulation schemes are effective to suppress the pathologicalβoscillations when control signals are added to these nuclei in Figure 4. Moreover, control signal is also a significant evaluation index, which is shown in the bottom of Figure 4. It is obvious that the four control signals fluctuate around zero.

### 3.3 Role of parameters *K* andτin control strategies

**FIGURE 5:**
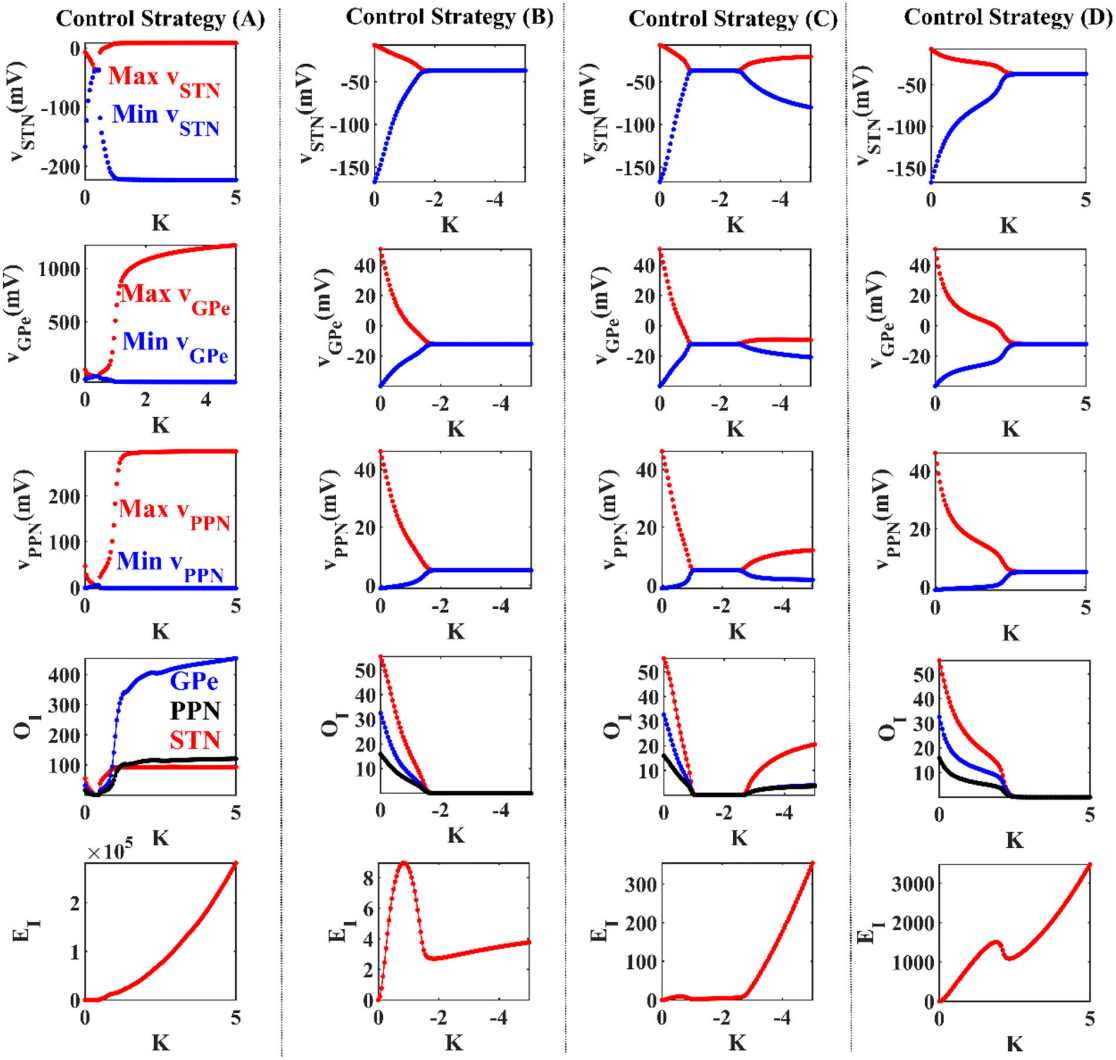
Role of the stimulation intensity *K* in controlling Parkinson's disease (PD) performance. All feedback stimulations are added att=1s. In rows ((1), (2), (3)), the bifurcation diagrams show the maximum and minimum values of local field potentials (LFPs) with respect to *K* for subthalamic nucleus (STN), globus pallidus (GPe), and pedunculopontine nucleus (PPN), respectively. In row (4), the average value of$O_{I}$for STN, GPe, and PPN as a function of *K* are given. In row (5), the energy consumption$E_{I}$of stimulation as a function of *K* is provided. Here, time delays are set toτ=25ms,τ=2ms,τ=4ms,τ=8msfor strategies (A–D), respectively.

As shown in Figure 5, we estimate the bifurcation diagrams for STN, GPe, PPN in four delayed feedback stimulation schemes to investigate the individual role of stimulation intensity *K*. In rows ((1), (2), (3)), the maximum and minimum of the$v_{i}\left(i\in \left\{\mathrm{STN},\mathrm{GPe},\mathrm{PPN}\right\}\right)$ of the last second are shown. All stimulation signals were added att=1s. We found that the positive *K* of$w_{m}\left(m\in \left\{\mathrm{STN},\mathrm{GPe},\mathrm{PPN}\right\}\right)$is effective in strategies (A) and (D), and the negative *K* of the$w_{m}$is effective in strategies (B) and (C). The primary factor is that STN and PPN are the excitatory population, and GPe is the inhibitory population. In the bifurcation diagram of Figure 5, it is obvious that the positive range of *K* for schemes (A) and (D) can suppress the oscillatory activity, while for schemes (B) and (C), only negative range can suppress. Moreover, in schemes (B) and (D), with the increasing of parameter *K*, the system only changes from the pathological state to the normal state. In schemes (A) and (C), the system produces pathological oscillation activities from the pathological state to the normal state with the increasing of parameter *K*, but it goes back to pathological state again when parameter *K* is large enough. Thus, the *K* is effective to suppress the oscillation activities in the right range. In addition, compared with the traditional scheme (A), the novel schemes (B‒D) can suppress oscillation activities in a wider range.

It shows the values of$O_{I}^{\mathrm{STN}}$,$O_{I}^{\mathrm{GPe}}$and$O_{I}^{\mathrm{PPN}}$in row (4) in Figure 5. The effective *K* varies in similar appropriate ranges. Therefore, new strategies (B‒D) are superior to the traditional strategy (A) in the variations of stimulation intensity *K*. Moreover, in row (5), we calculated the energy index of four delayed feedback stimulation schemes according to different values of *K*. In fact, stimulation schemes (B‒D) have a smaller energy expenditure with the larger range of the effective *K* compared with the traditional stimulation scheme (A).

Thus, we showed that oscillations activities in the system can be suppressed by adjusting the value of parameter *K*. As the parameter *K* increased, it could suppress the pathological state, but when it increased to a larger range, the system might strengthen the pathological oscillations. By comparing the robust performance for *K* and energy consumption, novel strategies (B‒D) with higher robustness and less energy consumption were more appropriate than strategy (A).

**FIGURE 6:**
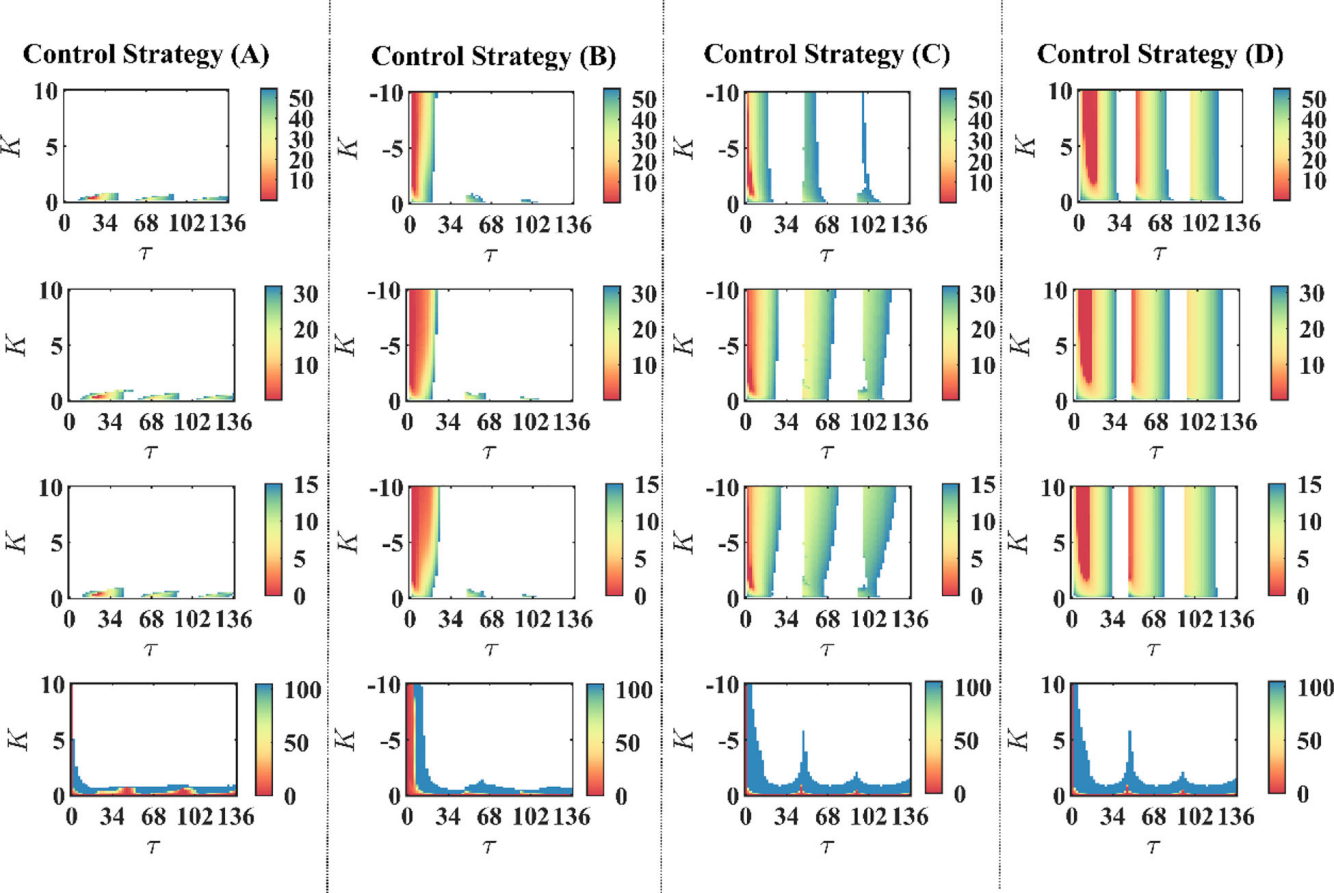
Effect of stimulation intensity *K* and time delayτon suppressing Parkinson's disease (PD) state. The oscillatory index$O_{I}^{STN}$,$O_{I}^{GPe}$,$O_{I}^{PPN}$and the energy consumption$E_{I}$of the stimulation are shown in (τ,K)panel. There are some oscillation suppression islands, and the white color region represents the large value without using delayed feedback control.

Similar to previous studies on parameter *K*, due to the importance of time delay in the delayed feedback algorithm design, we also studied the role of time delay in suppressing the oscillation activities. Figure 6 shows the oscillatory index$O_{I}$ and energy consumption$E_{I}$of STN, GPe, and PPN, which is worked by stimulation intensity *K* and time delayτ. In the 2‐D parameter panel, there are some oscillation suppression islands in the first three rows. As *K* andτvariation, the color of the 2‐D parameter panel denotes the variations of oscillatory index$O_{I}$. In addition, the white color region represents the huge oscillations with a large value of the$O_{I}$compared with the pathological state without control.

To observe the effect of four delayed feedback stimulation schemes, Figure 6 shows the$O_{I}$and $E_{I}$ when *K* andτboth changed. Here, we found three oscillation suppression islands, but these islands’ centers were different. Strategy (A) has a large of central time delay of 25 ms, and the central time delay shifts to 2, 4, and 8 ms for strategies (B), (C), and D), respectively. The reasons may be a self‐inhibitory connection in GPe, the addition of excitatory neurons PPN, and stimulating the nucleus with the LFPs of other nuclei. Therefore, strategies (B–D) speed up the propagation to suppress oscillation activities and cut down the time delay.

We found that stimulation schemes (B‒D) have a larger area of the first oscillation suppression islands than the traditional strategy (A) by observing the first three rows in Figure 6. Thus, considering the control theory robustness factor, these findings further indicated that strategies (B‒D) maybe more superior than strategy (A).

The last row also demonstrates the energy consumption of the four feedback stimulation schemes. In addition, blue regions show that the value of$E_{I}$is over or equal to 100. Our results demonstrated that schemes (B‒D) produce a larger area of energy consumption for suppressing the pathological oscillations compared with strategy (A). Therefore, we concluded that strategies (B–D) are more appropriate than strategy (A) because of the higher control strategy robustness and saving more energy.

## 4 DISCUSSION

The alpha and beta oscillations play an important role in PD research. In a recent study, alpha oscillations in PPN are significantly enhanced after levodopa administration in patients with PD, and its intensity is positively correlated with the improvement of gait in PD (Thevathasan et al., 2012). In addition, Thevathasan et al. (2012) recorded the LFP of PPN during walking and sitting in patients with PD. Alpha and beta oscillations were observed in the PPN, with alpha oscillations mainly in the caudal side of the PPN and beta oscillations mainly in the rostral part of the PPN. The experiment shows that stimulating different areas of PPN may have different therapeutic effects. Beta oscillations in PPN are controversial. Some researchers believe that they may have anti‐kinetic effects similar to beta oscillations in the basal ganglia, but Tsang et al. (2010) have obtained different results. Therefore, alpha and beta oscillations in PPN are important physiological oscillatory electrical activities, which need to be studied in the future.

The pathologic oscillations of PD include not only alpha and beta oscillations but also gamma oscillations. Gamma oscillations cause an inflexible state of neural activity that suppresses changes in normal motor and thus leading to PD states such as dyskinesia. Swann et al. (2016) proposed a new multi‐point long‐term recording of patients’ physiological data and found that gamma oscillations appeared in patients with PD with levodopa‐induced dyskinesia. Furthermore, Fraix et al. (2013) suggested that the stimulation of PPN can enhance patients’ alertness and that gamma oscillations are related to alertness, which may enhance patients’ locomotion. Alpha and gamma oscillations need to be further explored in the neural mass model, and our delayed feedback system will be able to control both alpha and gamma oscillations as well as beta oscillations.

There are still challenges to be overcome with PPN stimulation. Stimulating PPN nucleus is considered in some cases to stimulate the area of PPN. Because of internal PPN nucleus boundary ambiguity, the operation of electrode implantation will be difficult and may bring risks such as surgical infection (Molina et al., 2021; Thevathasan et al., 2018). Furthermore, PPN DBS was shown to improve freezing and falls, but improvements in other aspects of the bradykinesia, tremor, or rigidity may be limited (Ferraye et al., 2011). In future, much work needs to be done to understand the interaction of PPN and other neurons such as basal ganglia and clinical therapeutic effect of PPN stimulation. Although the patient data for PPN stimulation are limited, we still consider PPN as a potential stimulation target. Therefore, the development of PPN stimulation as a reliable clinical treatment requires the joint efforts of a large number of researchers.

Although we demonstrated delayed feedback stimulation in the neural mass model availability of control PD. However, the model and the control schemes still have some limitations. We only considered three main nuclei in our model, such as Str, Cor, and GPi, which need further consideration because of the simulation of the more realistic brain dynamic. The oscillatory activity can finally be suppressed fluctuating around zero by our simulated stimulation. But the index is idealized to show the effect of four control schemes, and the result of stimulating the patients will produce some bias. Hopefully, this study is helpful in improving the effect of the novel closed‐loop desynchronizing DBS system and paving a path toward the treatment of PD.

In the early stages of PD, the disease is usually treated with levodopa. Levodopa is a biological precursor of DA, which can be converted into DA when entering the human brain nervous system to supplement the shortage of DA (Baston et al., 2016). As the levodopa drug does not work well or the disease worsens, it is considered to implant electrodes at specific targets in the brain for stimulation (Eusebi et al., 2018; Rahimi et al., 2011). These additional variables affecting PD with motor and non‐motor symptoms should be added to supplement our model in future research. We will further quantify the association between levodopa levels and Parkinson's symptoms in clinical trials. Future work needs to find an appropriate dose of levodopa based on the stage and severity of PD using the neural mass model that can control the symptoms of the disease and reduce side effects. In addition, this model should be further studied to predict the moment when individuals need to increase the dose of levodopa or DBS stimulation, and when they need to decrease the dose.

Delayed feedback stimulation can help improve patient outcomes by providing more precise and effective stimulation, leading to better symptom control and quality of life. In the future, improving stimulus efficiency and increasing stimulus diversity should be considered.

First, determining the appropriate interphase gap which requires a large number of experiments to support plays an important role in DBS treatment. Interphase gap refers to the time interval between positive and negative electrical pulses in an electrical stimulation. The effectiveness and security of stimulation depend on the interphase gap time. It may cause interference between electrical pulse with short gap time and may reduce treatment effectiveness with long gap time. Stimulation pulses can get a better desynchronization effect by adjusting the interphase gap. Determining the proper interphase gap will require further exploration in the future.

Besides, delayed feedback technology includes linear delayed feedback (LDF) and nonlinear delayed feedback (NDF) stimuli, which are applied to different control scenarios. In this study, we used the typical linear delayed feedback control to stimulate the experiment. However, NDF with wide applicability and excellent robustness also effectively suppresses the synchronization, so we will further explore NDF for the treatment of PD. In addition, comparing with our control methods, these stimulation methods, such as adding stimulation when LFP exceeds a set threshold or synchronizing the stimulation signal with the neuronal activity cycle, are also well worth studying in the future.

## 5 CONCLUSION

An experimental study computationally tested and verified the effect of four delayed feedback stimulation strategies in modulating pathological oscillations based on the neural mass model involving STN, GPe, and PPN. The aim of this study was to improve the effect of stimulation pattern and enlarge the delayed feedback control parameter space.

We designed a neural mass model, including STN, GPe, and PPN, to control the pathological state. Considering the role of excitatory PPN and inhibitory GPe, we designed and tested three novel delayed feedback stimulation schemes compared with only stimulating the STN itself. The following conclusions may be drawn from the experimental results: first, the new designed neural mass model can reproduce the normal and pathological state. Second, by adjusting these appropriate parameters of time delayτand stimulation intensity *K*, it can be found that oscillation activities can be transferred to the normal state for the four delayed feedback stimulation schemes. Finally, three novel stimulation schemes can suppress the pathological state effectively, which have less energy consumption and higher control schemes robustness. Thus, the neural mass model provides a practicable means to research and suppress PD, and the three new stimulation schemes are more appropriate for suppressing PD than the traditional stimulation scheme in the future.

## CONFLICT OF INTEREST STATEMENT

The authors declare no conflict of interest.

### PEER REVIEW

The peer review history for this article is available at https://publons.com/publon/10.1002/brb3.3183.

## Data Availability

The data used to support the findings of this study are available from the paper.
